# Pressure-Induced Phase Transition and Mechanical Properties of Mg_2_Sr Intermetallics

**DOI:** 10.3390/ma9110902

**Published:** 2016-11-08

**Authors:** Haiyan Yan, Xingming Han, Baobing Zheng

**Affiliations:** 1 College of Chemistry and Chemical Engineering, Baoji University of Arts and Sciences, Baoji 721013, China; 2 Department of Computer Science, Baoji University of Arts and Sciences, Baoji 721016, China; xmh079@163.com; 3 College of Physics and Optoelectronics Technology, Nonlinear Research Institute, Baoji University of Arts and Sciences, Baoji 721016, China; scu_zheng@163.com

**Keywords:** intermetallics, phase transition, elastic anisotropy, electronic structure

## Abstract

A pressure-induced phase transition of Mg_2_Sr intermetallics from the low-pressure C14-type phase to an orthorhombic phase (space group *Cmcm*, *Z* = 4) at a high pressure of 21.0 GPa was firstly predicted using first-principles calculations combined with unbiased swarm structure searching techniques. The phase transition was identified as a first-order nature with a volume drop of 4.7%, driven by the softening of elastic behavior at high pressure. Further phonon calculations indicate that the newly predicted orthorhombic phase is dynamically stable at high pressure and ambient pressure. The mechanical properties including the elastic anisotropy of this orthorhombic phase were thus fully studied at ambient pressure. The elastic anisotropy behavior of this orthorhombic phase was investigated by the distributions of elastic moduli. The evidence of the bonding nature of Mg–Sr was also manifested by density of states (DOS) and electronic localization function (ELF) calculations.

## 1. Introduction

The study of Mg intermetallics has been attracting much attention because of their important applications in the automobile and aerospace industries [1,2]. Among the Mg-based intermetallics, the Mg–RE (RE = Ca, Sr, and Ba) system’s intermetallic compounds have emerged as promising candidate materials for transportation, aeronautical, and helicopters [3,4] and have thus generated significant interest over the past few decades. The binary phase diagrams of Mg–Ca, Mg–Sr, and Mg–Ba have been investigated for a long time, and different stable intermetallics have been found in these systems [5,6,7,8]. Due to their importance, extensive studies have been undertaken of crystal structures and lattice parameters [9,10,11], as well as thermodynamic properties [12,13] for these Mg-based intermetallics. In the Mg–Sr system is a typical Laves phase Mg_2_Sr, the heat formation of which was firstly measured by King and Kleppa [14] by means of tin solution calorimetry. In a recent work, Aljarrah and Medraj [15] reoptimized the Mg–Sr system in the CALPHAD approach considering all the available experimental data on the phase diagram and provided the crystallographic data, heat formation, and lattice parameters of four intermetallic compounds (Mg_2_Sr, Mg_38_Sr_9_, Mg_23_Sr_6_, and Mg_17_Sr_2_). The heat formation of Mg_2_Sr reported by Aljarrah and Medraj is in agreement with that of Yang et al. [16], who also investigated the elastic property and density of state (DOS) of this intermetallic phase. 

More recently, the mechanical properties, electronic structures, as well as thermodynamic properties of the Mg_2_Sr Laves phase under high pressure have been systematically investigated using the first-principles calculations by Mao et al. [17]. These extremely important results are significant to extend our knowledge to materials performance under extremely severe environments and will inevitably advance our understanding of high-pressure behaviors for other Mg intermetallics. The pressure-induced phase transition sequence of compounds such as Mg_2_Si has been determined with the aid of experimental and theoretical studies [18]. However, the peculiarity and the absence of further characterized high-pressure phases and related fundamental mechanical properties prompted our endeavor to investigate Mg_2_Sr intermetallics at a higher pressure. It is well known that high-pressure research leads to the identification of novel behavior of solids and the exploration of potential technological materials, since pressure can significantly alter the electronic bonding state to modify the physical properties, to induce the structural phase transition, or both. In addition, from the recent work for Mg_2_Sr [17], its elastic parameters exhibit a clear softening trend at an elevated pressure, especially for single crystal elastic constant *C*_44_, signifying its structural instability at high pressures. Therefore, we here performed extensive structure searches to explore the crystal structures of Mg_2_Sr over a range of pressures (0–50 GPa), based on a global minimization of free-energy surfaces merging ab initio total energy calculations via the particle swarm optimization technique [19]. Indeed, the pressure-induced transition into an orthorhombic phase (*Cmcm*, *Z* = 4) for Mg_2_Sr at 21.0 GPa was firstly predicted. First-principles calculations were then performed to investigate the crystal structures, mechanical, and electronic properties for this novel orthorhombic phase.

## 2. Computational Methods

The high-pressure structure searches of Mg_2_Sr were performed by the recently developed Crystal structure AnaLYsis by Particle Swarm Optimization (CALYPSO) package [19,20], which has perfectly predicted the crystal structures of a diverse variety of materials [21,22,23,24,25]. In more detail, the variable-cell structure predictions were executed at 0, 10, 25, and 50 GPa with systems containing 1–6 formula units (f.u.) in the simulation cell. Structural relaxation, total energy, and electronic structure calculations were mainly performed using the density functional theory with the Perdew–Burke–Ernzerhof generalized gradient approximation [26,27], as implemented in the VASP code [28]. The projector augmented wave (PAW) method [29] was used to describe the electron-ion interactions, with 3*s*^2^ and 4*s*^2^4*p*^6^5*s*^2^ treated as valence electrons for Mg and Sr, respectively. A kinetic energy cutoff of 400 eV for the plane-wave expansion and dense *k*-point with grid density of 2π × 0.03 Å^−1^ (Monkhorst–Pack scheme) [30] were used in the Brillouin zone integration. The enthalpy and electronic band structure were calculated using the tetrahedron method with Blöchl corrections. Finite displacement method [31], which is based on first-principles calculations of total energy, Hellman–Feynman forces, and the dynamical matrix as implemented in the PHONOPY package to calculate the phonon spectra. The supercell of 3 × 3 × 3 original cell containing 162 atoms was adopted in the phonon calculations for Mg_2_Sr. The strain-stress method was applied to calculate the single crystal elastic constants, and the polycrystalline bulk modulus and shear modulus were thus derived from the Voigt–Reuss–Hill averaging scheme [32].

## 3. Results and Discussion

**Figure 1:**
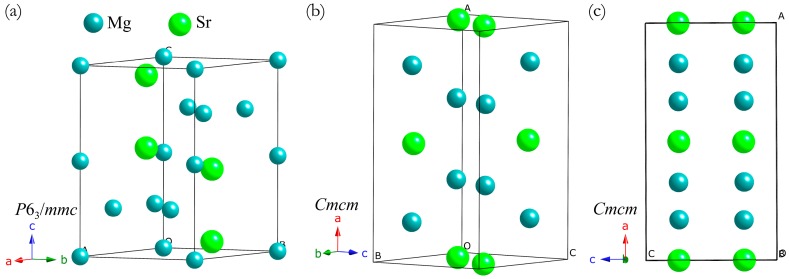
Crystal structures of Mg_2_Sr. (**a**) Low-pressure C14-type phase; (**b**) The predicted high-pressure orthorhombic *Cmcm* phase; (**c**) The projection of orthorhombic *Cmcm* phase on *ac*-plane. The large and small balls denote Sr and Mg atoms, respectively.

**Figure 2:**
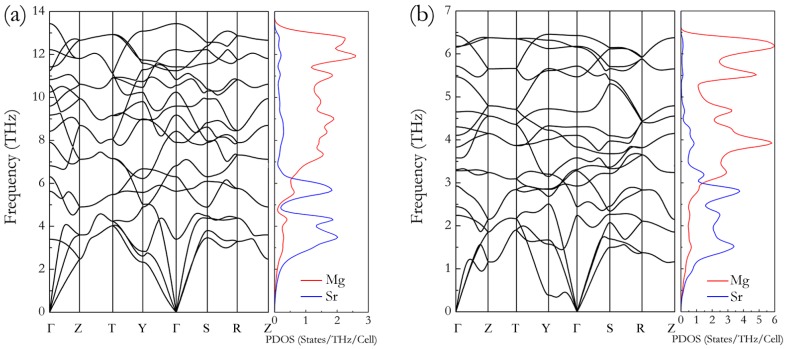
Phonon curves of the orthorhombic *Cmcm* phase at (**a**) 50 GPa and (**b**) 0 GPa.

Using the CALYPSO package, at pressures of 0 and 10 GPa, the experimental C14-type phase of Mg_2_Sr with space group *P*6_3_/*mmc* (*Z* = 4, see Figure 1a) was successfully reproduced from a global structure search, validating our method adopted here. In Figure 1a, the C14-type phase contains twelve atoms per unit cell with eight Mg atoms occupying the 2*a* and 6*h* positions and four Sr atoms occupying the 4*f* positions. At ambient pressure, the optimized equilibrium structural parameters for C14-type phase are *a* = 6.456 Å, *c* = 10.45 Å, and *V*_0_ = 377.236 Å^3^, which are all in good agreement with the available experimental data (*a* = 6.484 Å, *c* = 10.451 Å, and *V*_0_ = 380.5 Å^3^) [33] and theoretical values [17]. In addition, the theoretical equations of states (EOS) of Mg_2_Sr studied here were determined by fitting the total energies as a function of the volumes based on the Birch–Murnaghan EOS. The obtained bulk modulus *B*_0_ and its pressure derivative *B*_0_′ of Mg_2_Sr (25.946 GPa and 3.644) also agree well with the previous theoretical values [17]. The success in the prediction of experimental C14-type phase gives us confidence to further explore the high-pressure phases of Mg_2_Sr. For higher pressures at 25 and 50 GPa, a novel orthorhombic phase with *Cmcm* space group (*Z* = 4, hereafter denoted as HP, see Figure 1b) was discovered for Mg_2_Sr as the most energetically stable. At 30 GPa, the optimized lattice parameters of HP-Mg_2_Sr are *a* = 8.585 Å, *b* = 5.399 Å, and *c* = 4.754 Å, with Mg and Sr atom occupying the 8*g* (0.329, 0.366, 0.25) and 4*c* (0, 0346, 0.25) positions, respectively. The projection of the orthorhombic HP phase on the *ac*-plane is presented in Figure 1c, from which the HP phase possesses alternative stacking of double Mg and single Sr layers along the *a*-direction. Each Mg ion is coordinated with five Sr ions, and each Sr ion is coordinated with ten Mg ions. Physically, the phonon is a strict measure for structural dynamic stability. We thus carefully checked the phonon frequency curves of HP-Mg_2_Sr at 50 GPa (Figure 2a) and 0 GPa (Figure 2b). In Figure 2, there is no imaginary phonon frequency in the entire Brillouin zone, i.e., the HP-Mg_2_Sr is dynamically stable at high and ambient pressures. The lower frequencies of the phonon density of states are dominated by lattice dynamics of heavy Sr atoms and higher frequencies by light Mg atoms.

**Figure 3:**
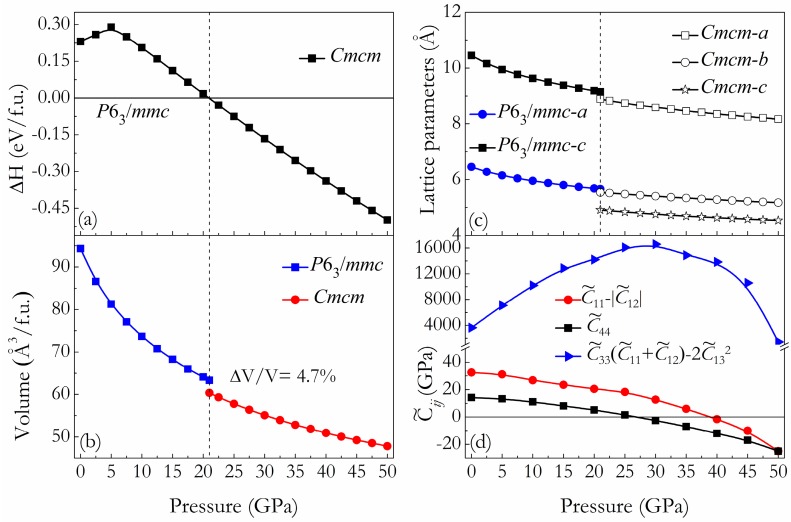
(**a**) Enthalpy differences of high-pressure *Cmcm* phase relative to the low-pressure *P*6_3_/*mmc* phase as a function of pressure; (**b**) The calculated volumes as a function of pressure for the *P*6_3_/*mmc* and *Cmcm* phases; (**c**) The calculated lattice parameters as a function of pressure for the *P*6_3_/*mmc* and *Cmcm* phases; (**d**) The calculated $\tilde{C_{44}}$, $\tilde{C_{11}}-\left|\tilde{C_{12}}\right|$ and $\tilde{C_{33}}\left(\tilde{C_{11}}+\tilde{C_{12}}\right)-\tilde{2{C_{13}}^{2}}$ under different isotropic pressures.

To determine the phase transition pressure point of Mg_2_Sr, the enthalpy differences curves of the predicted HP phase relative to the C14-type phase are presented in Figure 3a. We optimized these two structures at many more pressure points up to 50 GPa with certain pressure intervals. Figure 3a confirms that the predicted HP phase becomes more stable than the experimental C14-type phase above 21.0 GPa, where the current experimental techniques are readily accessible. Meanwhile, as shown in Figure 3b, one can see that the C14-type → HP phase transition is first-order with a clear volume contraction of 4.7%. Such an obvious volume reduction at the transition is easy to detect in a high pressure X-ray powder diffraction experiment. The pressure dependence of lattice constants for two phases are also plotted in Figure 3c to complement further experiments. The pressure dependence of elastic constants is a very important indicator of the mechanical stability of crystal. We first calculated the single crystal elastic constants of C14-type phase at ambient pressure, as tabulated in Table 1 together with the theoretical results for comparisons. From Table 1, one can see that the calculated elastic constants and the derived Hill elastic moduli of Mg_2_Sr are in excellent agreement with previous theoretical results [16,17]. Furthermore, the mechanical stability for the hexagonal crystal under isotropic pressure is provided in [34]. It requires the following conditions: $\tilde{C_{44}}>0,\;\tilde{C_{11}}-\left|\tilde{C_{12}}\right|>0,$ and $\tilde{C_{33}}\left(\tilde{C_{11}}+\tilde{C_{12}}\right)-\tilde{2{C_{13}}^{2}}>0,$ where $\tilde{C_{\alpha \alpha }}=C_{\alpha \alpha }-P$ (*α* = 1, 3 and 4), $\tilde{C_{12}}=C_{12}+P$, and $\tilde{C_{13}}=C_{13}+P$ Thus, we performed calculations on the pressure dependence of elastic constants for the C14-type phase. As shown in Figure 3d, the values of $\tilde{C_{33}}\left(\tilde{C_{11}}+\tilde{C_{12}}\right)-\tilde{2{C_{13}}^{2}}$ for C14-type phase are all positive at the considered pressure range. However, one can see that the values of both $\tilde{C_{44}}$ and $\tilde{C_{11}}-\left|\tilde{C_{12}}\right|$ show a softening trend, and the $\tilde{C_{44}}$ first drops to zero at about 26.6 GPa. These results suggest that the C14-type phase is mechanically unstable when *P* > 26.6 GPa under high pressure. We thus conclude that there must be a structural phase transition occurring in the pressures according to relations of the mechanical stability under isotropic pressure, as suggested by Karki et al. [35] and Wang et al. [36]. These behaviors further confirmed the accuracy of the predicted phase transition pressure of Mg_2_Sr, although the obtained transition pressure (26.6 GPa) is relative larger than that (21.0 GPa) obtained from the enthalpy differences curves of Figure 3a.

**Figure 4:**
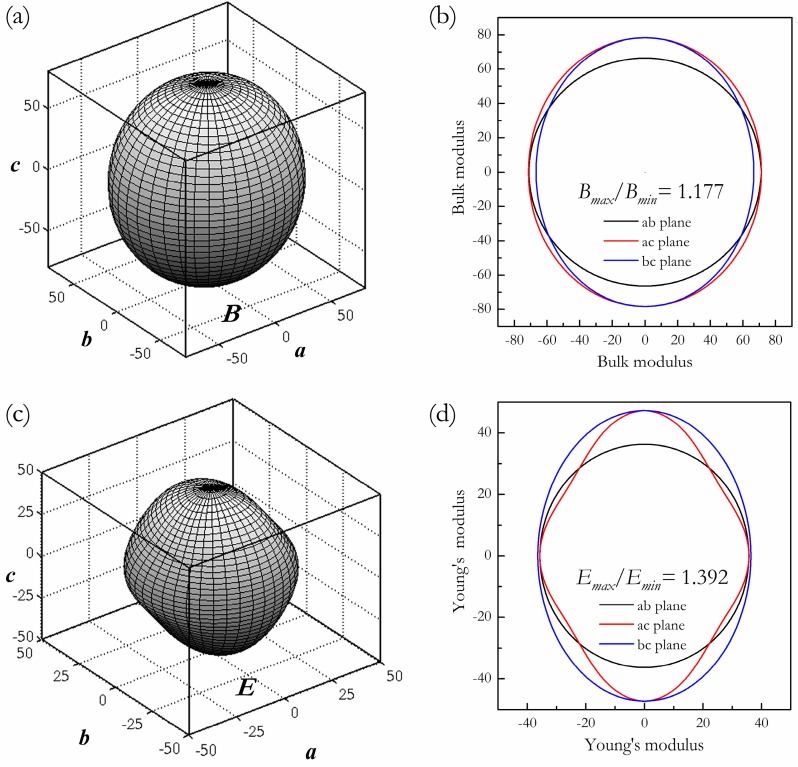
3D surface representation of the bulk modulus and Young’s modulus for the *Cmcm* phase (**a**,**c**). The projections of the bulk modulus and Young’s modulus within three main crystal planes (**b**,**d**).

**Figure 5:**
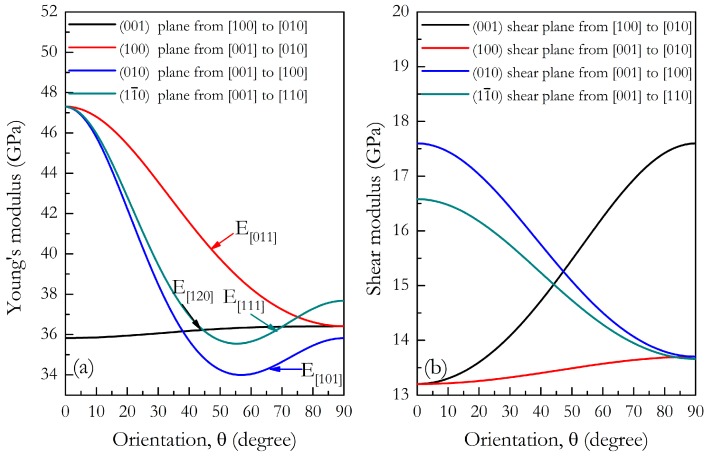
Orientation dependence of Young’s modulus (**a**) and orientation dependence of the shear modulus (**b**) in *Cmcm*-Mg_2_Sr.

Compared with the experimental C14-type phase, using the same method mentioned above, the mechanical properties (including the elastic stability, incompressibility, rigidity, and elastic anisotropy) of this predicted HP phase were fully studied at ambient pressure. The resulting single crystal elastic constants and the derived Hill elastic moduli are listed in Table 1. The mechanical stability of the HP phase satisfies the Born–Huang criterion for an orthorhombic crystal [*C*_11_ > 0, *C*_44_ > 0, *C*_55_ > 0, *C*_66_ > 0, *C*_11_*C*_22_ > *C*_12_^2^, *C*_11_*C*_22_*C*_33_ + 2*C*_12_*C*_13_*C*_23_ − *C*_11_*C*_23_^2^ − *C*_22_*C*_13_^2^ − *C*_33_*C*_12_^2^ > 0] [37], thus confirming that this HP phase is mechanically stable at ambient conditions. Moreover, the obtained elastic constants possess the trend of *C*_11_ ≈ *C*_22_ < *C*_33_, indicating that the bonding between nearest neighbors along the {001} planes is stronger than those along the {100} and {010} planes. Both the calculated elastic moduli of HP and C14-type phases are lower than those experimental data of pure Mg metal (*B* = 36.9 GPa, *G* = 19.4 GPa, and *E* = 49.5 GPa) [38]. Traditionally, and for ease of manipulation, the elastic properties of an anisotropic material were replaced by those of an “equivalent” isotropic material. Essentially, all the known crystals are elastically anisotropic, and the anisotropy of elasticity is an important implication in engineering science and crystal physics, such as microcracks, anisotropic plastic deformation, and elastic durability. To intuitively illustrate the elastic anisotropy of this predicted orthorhombic phase, the directional dependence of elastic moduli were systematically investigated. The calculations of elastic moduli crystal orientation dependence conducted here are similar to our previous studies [39]. Executing the appropriate coordinate system transformations for the compliances allows for the determination of the variation of bulk modulus *B*, Young’s modulus *E*, and shear modulus *G* with crystallographic direction, [*uvw*], for a given crystallographic plane, (*hkl*), containing these directions, (i.e., *B*_[*uvw*]_, *E*_[*uvw*]_, and *G*_(*hkl*)[*uvw*]_) are obtained. For orthorhombic HP-Mg_2_Sr, the bulk modulus *B* and Young’s modulus *E* can be expressed as

(1) $$B^{-1}=\left(s_{11}+s_{12}+s_{13}\right)\alpha^{2}+\left(s_{12}+s_{22}+s_{23}\right)\beta^{2}+\left(s_{13}+s_{23}+s_{33}\right)\gamma^{2},$$

(2) $$E^{-1}=s_{11}\alpha^{4}+s_{22}\beta^{4}+s_{33}\gamma^{4}+2s_{12}\alpha^{2}\beta^{2}+2s_{23}\beta^{2}\gamma^{2}+2s_{13}\alpha^{2}\gamma^{2}+s_{44}\beta^{2}\gamma^{2}+s_{55}\alpha^{2}\gamma^{2}+s_{66}\alpha^{2}\beta^{2},$$

where *α*, β, and γ are the direction cosines of [*uvw*] direction, and *s*_11_, *s*_22_, etc. are elastic compliance constants given in [40]. The shear modulus *G* on the (*hkl*) shear plane with shear stress applied along [*uvw*] direction is given by

(3) $$G^{-1}=4s_{11}{\alpha_{1}}^{2}{\alpha_{2}}^{2}+4s_{22}{\beta_{1}}^{2}{\beta_{2}}^{2}+4s_{33}{\gamma_{1}}^{2}{\gamma_{2}}^{2}+8s_{12}\alpha_{1}\alpha_{2}\beta_{1}\beta_{2}+8s_{23}\beta_{1}\beta_{2}\gamma_{1}\gamma_{2}+\;8s_{13}\alpha_{1}\alpha_{2}\gamma_{1}\gamma_{2}+s_{44}{\left(\beta_{1}\gamma_{2}+\beta_{2}\gamma_{1}\right)}^{2}+s_{55}{\left(\alpha_{1}\gamma_{2}+\alpha_{2}\gamma_{1}\right)}^{2}+s_{66}{\left(\alpha_{1}\beta_{2}+\alpha_{2}\beta_{1}\right)}^{2},$$

where (α_1_, β_1_, γ_1_) and (α_2_, β_2_, γ_2_) are the direction cosines of the [*uvw*] and [*HKL*] directions in the coordinate systems, where the [*HKL*] denotes the vector normal to the (*hkl*) shear plane. Three-dimensional (3D) surface representations showing the variation of the bulk modulus and Young’s modulus are plotted in Figure 4a,c, and three crystal plane projections of the directional dependence on the bulk modulus and Young’s modulus are given in Figure 4b,d for comparison. From Figure 4a,c, one can see that this HP phase exhibits a weak elastic anisotropy, for its bulk modulus and Young’s modulus distributions with the nonspherical nature. Compared with the in-plane anisotropy in *ab* and *bc* planes, a relatively clear in-plane elastic anisotropy in the *ac* plane is revealed for both the bulk modulus (*B_max_*/*B_min_* = 1.177) and Young’s modulus (*E_max_*/*E_min_* = 1.392). Like the elastic constants, Young’s modulus *E* measures the resistance against uniaxial tension, and shear modulus *G* describes the resistance of a material to shape change. In order to gain a better understanding of the origin of the changes in Young’s modulus and the shear modulus along different directions for further engineering applications, we next determined the detail orientation dependence on Young’s modulus and the shear modulus when the tensile axis is within specific planes according to the Equations (2) and (3). In Figure 5a, the ordering of Young’s modulus as a function of the principal crystal tensile [*uvw*] for HP-Mg_2_Sr is *E*_[001]_ > *E*_[011]_ > *E*_[110]_ > *E*_[010]_ > *E*_[111]_ ≈ *E*_[120]_ > *E*_[100]_ > *E*_[101]_. Compared with the other three crystal planes, the change of Young’s modulus in the (001) plane for the quadrant of directions [*uvw*] between [100] (θ = 0°) and [010] (θ = 90°) is flat, which accords well with the smallest anisotropy within *ab* planes (see Figure 4d). Similarly, the orientation dependence of the shear modulus on the stress directions within the four main crystal planes were also conducted, as plotted in Figure 5b. For the (100) shear plane with the shear stress direction rotated from [100] to [010], the direction cosines are α_1_ = cosθ, β_1_ = sinθ, γ_1_ = 0, α_2_ = β_2_ = 0, and γ_2_ = 1, where θ is the angle between the [100] and shear stress direction. From Equation (3), one can deduce the shear modulus expressed as *G*^−1^ = *s*_55_ + (*s*_44_ − *s*_55_) sin^2^θ. For HP-Mg_2_Sr, *s*_44_ < *s*_55_, the shear modulus is the largest along [010] and is the smallest along [001]. Among these four main crystal planes, it should be noted that the shear moduli within the (100) shear plane are the smallest with its minimum (*G*_(100)_[001] = 13.2 GPa) and maximum (*G*_(100)_[010] = 13.7 GPa) values, and are almost independent of any shear directions. Thus, the (100) shear plane may be viewed as the cleavage plane of HP-Mg_2_Sr. 

**Figure 6:**
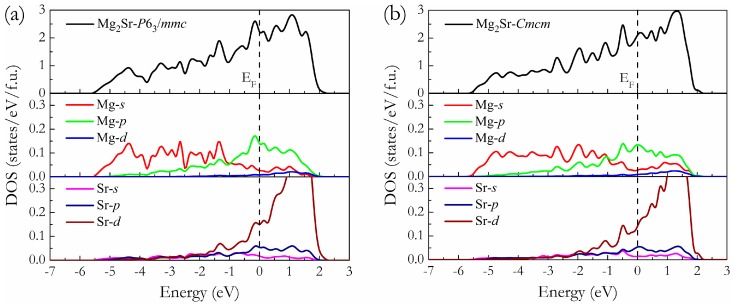
Calculated t-DOS and p-DOS of the *P*6_3_/*mmc* phase at 0 GPa (**a**) and those of the *Cmcm* phase at 0 GPa (**b**). The vertical dash line is the E_F_.

**Figure 7:**
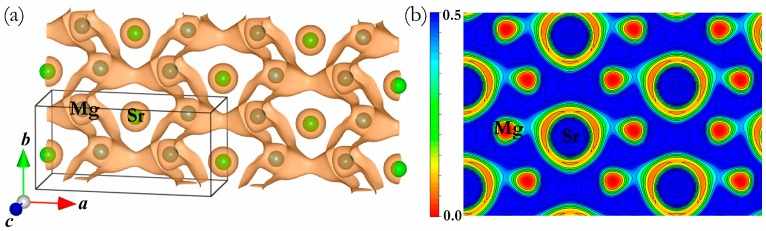
(**a**) 3D ELF distributions (ELF = 0.5) of the *Cmcm* phase at 0 GPa; (**b**) Contours of ELF for the *Cmcm* phase on (001) plane.

To understand the bonding characteristics of this new predicted intermetallic phase on a fundamental level, the total and partial density of states (t-DOS and p-DOS) of C14-typeand HP phase were calculated at 0 GPa, as shown in Figure 6a,b, where the vertical dash lines are the Fermi level (E_F_). From the t-DOS curves, one can see that both phases exhibit a clear metallic nature characterized by the evidence of the finite electronic DOS at the E_F_. Based on the inspections of the p-DOS of the C14-type and HP phases, their bonding states below E_F_ were found to be mainly dominated by a mixture of Mg–*s* and Mg–*p* states as well as Sr–*d* states. Meanwhile, the electrons of Mg–*p* have a significant hybridization with Sr–*d* states from about −3 eV up to E_F_, signifying a strong Mg–Sr covalent bonding nature. Apart from the covalent bonding feature of the HP phase, the ionic bonding nature of Mg and Sr atoms is also revealed by the electronic localization function (ELF) calculations at 0 GPa. For the selected 3D ELF distributions (ELF = 0.5) in Figure 7a, the high electron localization locates at the Mg sites rather than the Sr sites, reflecting the ionicity of the Mg–Sr bond. Meanwhile, one can note that the contours of the ELF domains on the (001) plane show nearly identical Mg–Sr ionic features at about ELF = 0.4 (see Figure 7b).

## 4. Conclusions

To conclude, the pressure-induced phase transition of Mg_2_Sr was predicted using a particle swarm optimization algorithm in crystal structure prediction. An orthorhombic high-pressure phase (space group *Cmcm*, *Z* = 4) of Mg_2_Sr was uncovered at 21.0 GPa. The transition of low-pressure C14-type phase to this orthorhombic HP phase was characterized as a first-order nature, driven by the softening of elastic behavior at high pressure. The elastic anisotropy of the predicted orthorhombic phase was demonstrated by the orientational distributions of the elastic moduli. The shear modulus is found to be the smallest within the (100) crystal plane, which may be viewed as its cleavage plane. Detailed analyses of the DOS and ELF reveal that the chemical bonding in the orthorhombic phase is a complex mixture of covalent and ionic characters.

## Figures and Tables

**Table 1:** Calculated elastic constants *C_ij_* (GPa), bulk modulus *B* (GPa), shear modulus *G* (GPa), Young’s modulus *E* (GPa), and Poisson’s ratio ν for Mg_2_Sr.

Mg_2_Sr	Source	*C*_11_	*C*_22_	*C*_33_	*C*_44_	*C*_55_	*C*_66_	*C*_12_	*C*_13_	*C*_23_	*B*	*G*	*E*	ν
C14-type	Present	49.9	-	56.9	14.2	-	-	17.4	10.2	-	25.8	16.6	41.0	0.235
	Theory ^a^	43.8	-	57.2	12.4	-	-	19.8	10.6	-	25.2	13.8	36.1	0.360
	Theory ^b^	43.7	-	56.5	12.4	-	-	20.1	11.9	-	25.4	13.7	34.8	0.230
HP	Present	43.1	42.9	52.0	17.6	13.2	13.7	15.7	12.6	10.7	24.0	15.3	37.9	0.237

^a^ Ref. [34]; ^b^ Ref. [17].
